# Balancing ethical and practical dilemmas: feasibility of a cluster randomised internal pilot trial of Teaching Recovery Techniques with accompanied refugee children in Sweden

**DOI:** 10.1186/s40814-025-01753-y

**Published:** 2026-01-02

**Authors:** Sandra Gupta Löfving, Farah Alsaqa, Anna Sarkadi, Elin Inge, Anna Pérez-Aronsson, Antónia Tökés, Georgina Warner

**Affiliations:** Child Health and Parenting (CHAP), Department of Public Health and Caring Sciences, Uppsala University, BMC, Husargatan 3, Box 564, 751 22 Uppsala, Sweden

**Keywords:** Teaching Recovery Techniques, Post traumatic stress disorder, Accompanied refugee children, Randomised controlled trial, Feasibility

## Abstract

**Background:**

Teaching Recovery Techniques (TRT) is a brief psychosocial intervention designed to reduce symptoms of post-traumatic stress among children. To strengthen the evidence base for TRT, a nationwide multisite cluster RCT of TRT with accompanied refugee children was planned in Sweden, including an internal pilot with the primary objectives of assessing screening, recruitment, attendance, and retention. Secondary objectives were to consider the feasibility of randomisation, the suitability of the questionnaires employed in the main RCT, and intervention acceptability.

**Methods:**

Accompanied refugee children aged 8 to 17 years, who arrived in Sweden within the last 5 years and screened positive for symptoms of post-traumatic stress, were allocated to the intervention or waitlist arm using non-blinded cluster randomisation. Pre- and post-measurements were conducted at baseline (T1) and after 8 weeks (T2). Success criteria for the pilot were (i) at least 50% of those referred for participation meet the screening cut-off for post-traumatic stress; (ii) 28 eligible children recruited in the first three months; (iii) at least 50% of those randomised to intervention attending one of the five core sessions; and (iv) at least 50% of those screened at T1 complete the T2 data collection. To get a deeper understanding of the acceptability of the intervention, 11 semi-structured interviews were conducted with refugee children. The interviews were transcribed and analysed using thematic analysis.

**Results:**

A change in recruitment strategy from referral to broader screening resulted in 44% meeting the cut-off for post-traumatic stress and, partly due to the COVID-19 pandemic, only five clusters (3 intervention, *n* = 11; 2 waitlist control, *n* = 11) were recruited over 12 months. However, 64% of those randomised to the intervention arm attended at least one of the five core intervention sessions, and 91% were retained at T2 data collection. The qualitative inquiry confirmed the acceptability of the intervention and the need for tools to address trauma symptoms but raised concerns about stigma related to both screening and participation.

**Conclusions:**

Although the planned RCT was deemed not feasible due to low recruitment rates, this pilot study gave insight into important practical and ethical considerations. Adjustments to information, screening, and recruitment may improve the likelihood of a successful RCT. Adopting a strength-based approach when introducing a mental health intervention to refugee children is recommended; not only may this reduce stigma, but it may actively shift from a deficit-focused discourse.

**Trial registration:**

ISRCTN17754931. Prospectively registered on 4th June 2019. https://doi.org/10.1186/ISRCTN17754931

**Supplementary Information:**

The online version contains supplementary material available at 10.1186/s40814-025-01753-y.

## Key messages regarding the feasibility

- The recruitment goal was not reached; However, attendance and retention rates indicated good acceptability of the intervention and study design.
- Qualitative inquiry confirmed the acceptability of the intervention and the need for tools to address trauma symptoms in refugee children. While the group setting fostered belonging for some, others felt that group dynamics limited their participation, highlighting the need to consider stigma and social hierarchies in school-based interventions.
- The internal pilot study identified ethical and practical dilemmas, underscoring the need for cultural humility and sensitivity in research with refugee children. Future RCTs should evaluate the separation of accompanied and unaccompanied children, assess screening purposes and methods, and ensure meaningful engagement with refugee advisors throughout the research process.

## Background

**Fig. 1:**
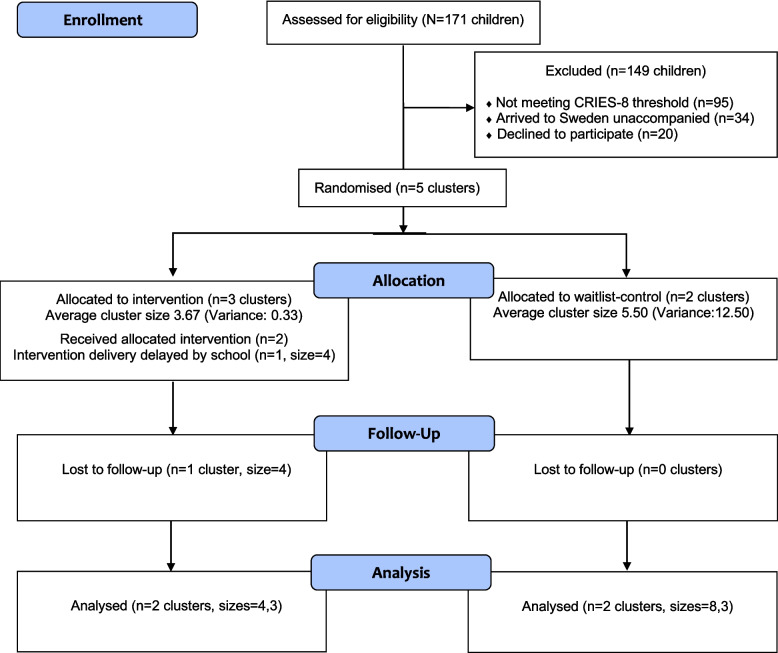
CONSORT flow diagram, presenting the flow of participants and clusters through each stage of the trial. Allocation, follow-up, and analysis were conducted at the cluster level. Average cluster sizes and their statistical summaries are reported

During the past decade, the number of refugees globally has tripled and reached unprecedented figures [1]. The psychological impact of trauma and migration on child mental health is well established. The situational vulnerabilities experienced before migration, during transit, and after resettlement have resulted in a high prevalence of post-traumatic stress symptoms in refugee children [2–4]. A review of the mental health of refugee children living in the Global North found a prevalence of post-traumatic stress disorder (PTSD) ranging from 19 to 54% [4]. Despite the high prevalence of psychological distress among this population, a review of mental health care utilisation among refugees in Europe reveals low rates of service use [5], and the mental health treatment gap for populations affected by these experiences remains deeply concerning [6].

The World Health Organization has identified various barriers at multiple levels that restrict access to care, including legislative exclusions and the absence of culturally responsive and linguistically appropriate services [6]. Additionally, factors such as the stigma surrounding mental health, distrust of authorities, and the overwhelming demands of resettlement may further discourage refugees from seeking the mental health support they need [7, 8].

The mental health service gap makes interventions situated in the community, particularly schools, important for refugee children. Schools may represent a familiar, accessible setting, potentially reducing stigma [9]. In addition, schools can facilitate consistent engagement, early identification of issues, and ongoing support for refugee children, making the setting an especially suitable environment for addressing children’s mental health needs [10].

Teaching Recovery Techniques (TRT) is a community-based group intervention designed by the Children and War Foundation in Norway, with the aim of mitigating symptoms of post-traumatic stress in children aged 8 to 18 years who have experienced war or natural disasters [11]. Developed to maximise reach, TRT prioritises a broader provision of ‘light-touch’ post-traumatic stress support over intensive treatment for a limited number of individuals.

TRT aligns with the core principles of Trauma-Focused Cognitive Behavioural Therapy (TF-CBT), the treatment of choice for children with post-traumatic stress [12]. TRT uses a task-shifting approach by training non-specialists to deliver the intervention after brief training. The intervention consists of five 90-min sessions for children and two additional sessions for caregivers. TRT’s five sessions target core trauma symptoms such as hyperarousal, avoidance, and intrusion, offering psychoeducation on typical trauma responses, including anxiety, hypervigilance, nightmares, and flashbacks [11]. This approach assists children in recognising that their reactions are common and legitimate, potentially alleviating feelings of isolation and shame [13].

Additionally, TRT presents relaxation techniques developed to calm the nervous system and reduce muscle tension as well as creates a mental ‘safe space’ by visualisation techniques to access when feeling stressed or overwhelmed. Furthermore, TRT introduces cognitive restructuring by challenging and replacing negative beliefs often following trauma such as safety, trust, and self-worth. Finally, TRT offers a session on exposure and setting future goals, the latter to highlight children’s resilience and enable envisioning a positive future [11].

The two caregiver sessions offer psychoeducation on trauma and trauma responses, along with a concise overview of the tools introduced and practised by the children. These sessions aim to prepare parents to assist their children in applying the techniques at home and to motivate parents to use the techniques for their own well-being.

While developed in the Global North, TRT was originally intended to meet the mental health needs of children residing in the Global South, where resources can be scarce. Randomised controlled trials (RCTs) in the Global South, particularly in Palestine, have found that children participating in TRT showed reductions in post-traumatic stress, depressive symptoms, distress, and grief, as well as improvements in psychosocial well-being [14–16]. An RCT focused on Palestinian children in the West Bank was paused after the Hamas attack and subsequent genocide in Gaza, but was later redesigned as a pre/post-test during this period of violence, yielding promising results [17]. Similarly, an RCT with Syrian refugee children in Lebanon showed enhanced mental health benefits when extra sessions for caregivers were included [18]. Additionally, a pilot trial among children in Brazilian favelas exposed to violence indicated reduced symptoms of post-traumatic stress and depression, confirming the feasibility of an RCT [19].

To address the urgent psychological needs of refugee children who fled to Sweden in response to the global political upheaval in 2015, TRT was implemented in Sweden as part of a stepped-care model [13]. This initiative aimed to fill the gap in Swedish mental health services, which were unable to meet the rising demand. A ‘getting to know each other session’ was offered prior to the core TRT sessions as well as a ‘follow-up session’ to consolidate learning. The scale-up model employed for TRT in Sweden aligns with a distribution network pathway [20]. This approach facilitates rapid dissemination of the intervention; however, it limits the distributing organisation’s oversight of local implementation. Consequently, this model may have posed challenges for group leaders, both in seeking timely support and in fostering mutual support networks, due to the absence of a central coordinating body [21]. Evident features among the sites maintaining TRT are collaboration with other stakeholders involved with refugees, such as civil society, churches, and government organisations, the use of interpreters as cultural brokers, and delivering TRT at locations where refugee children and young people reside [21].

Besides Sweden, TRT has been implemented for refugee children in the United Kingdom [22] and Norway [23]. However, there remains a need to strengthen the evidence base for its effectiveness for refugee children with trauma symptoms in the Global North. In Sweden, two national RCTs were launched to evaluate TRT for refugee children and young people: the SUPpORT study [24] focusing on unaccompanied minors, and the ASsIST study [25], addressing accompanied refugee children. The rationale for conducting two separate trials stemmed primarily from differences in mental health status and living circumstances between unaccompanied and accompanied refugee children. Unaccompanied youth, lacking parental protection, are more frequently exposed to severe traumas, including torture, sexual assault, and kidnapping, in comparison to their accompanied peers [26–28]. Unaccompanied refugee youth must navigate the complex asylum-seeking process without caregivers. In Sweden, several legislative changes have significantly impacted these unaccompanied refugee youth [29], making their circumstances different from those of accompanied children. Consequently, unaccompanied youth exhibit higher rates of PTSD, a finding observed both in Sweden and internationally [30, 31]. Additionally, the trials required separate recruitment strategies, as a significant portion of unaccompanied youth were to be recruited from residential care facilities specifically designated for this population.

The present internal pilot study was embedded within the ASsIST trial [25]. A cluster design was adopted because public advisors involved in the main trial indicated that individual-level randomisation was unacceptable, describing it as akin to a ‘lottery of the asylum’. This approach was also chosen to preserve existing friendship groups. The primary objectives of the internal pilot study were to assess the feasibility of the following:

(i) Screening process
(ii) Recruitment rate
(iii) Intervention attendance
(iv) Trial retention

The success criteria for the internal pilot were: (i) at least 50% of those referred for participation meet the screening cut-off (≥ 17) on the 8-item Children’s Revised Impact of Events Scale (CRIES-8) [32]; (ii) at least 28 eligible children were recruited in the first 3 months; (iii) at least 50% of those randomised to intervention attended one of the five core sessions; and (iv) at least 50% of those screened at T1 completed the T2 data collection. The target sample size for the internal pilot was 28 eligible children, which was derived from theoretical optimal values based on the power calculation for the main trial [33]. As the main trial was powered at 80% to detect a medium (0.50) effect size [25], a pilot study with 14 participants per arm was recommended when applying the inflation method [33]. Secondary objectives were to consider the (i) feasibility of randomisation; (ii) suitability of the questionnaires employed in the main RCT; and (iii) intervention acceptability.

## Methods

### Trial design

A two-arm cluster randomised waitlist-control pilot trial of TRT was conducted employing a 1:1 randomisation ratio between intervention and control arms. The study was planned as an internal pilot of the ASsIST trial [25]. Although the main trial collected data at three time points, this manuscript only includes the first two, as the internal pilot and associated success criteria were structured around them. A process for Decision-making after Pilot and Feasibility Trials (ADePT) [34] was selected to support systematic decision-making in moving forward with the trial. Ethical approval for the study was obtained from the Regional Ethical Review Board in Uppsala (Ref.2018/382) on 24th February 2019, and the trial was prospectively registered at the ISRCTN Registry (Ref. ISRCTN17754931).

The main trial [25] was designed to include participants aged 8 to 17 years. However, in this internal pilot, a small number of participants aged 18 years were randomised. This constitutes a deviation from the prespecified eligibility criteria. Potential explanations for this deviation include participants reaching the age of 18 between initial screening and randomisation, inadvertent application of criteria from a parallel trial (SUPpORT) in which participants aged 14–20 years are eligible [24], or a deliberate ethical decision to avoid excluding individuals who had only marginally exceeded the age threshold.

### Screening and recruitment

Screening took place at secondary and upper-secondary schools in four medium-sized Swedish cities. TRT sessions were delivered at the schools, with each group led by two TRT-trained group leaders, with an interpreter when necessary. Children were screened for post-traumatic stress symptoms using the CRIES-8 [32]. Those with a total score of 17 or above were eligible to participate in the study. The intention was for children to be referred either by self-referral or by parents or counsellors. However, at some sites, children were screened in a classroom setting. The inclusion and exclusion criteria, as well as the procedure of informed consent, are described in detail in the trial protocol [25]. Written consent was obtained from all participants 15 years and above; for those under 15 years, written assent was obtained, as well as written consent from caregivers.

### Randomisation

Cluster randomisation, by TRT group, was planned with an anticipated average cluster size of 6 participants. The clusters were organised by friendship groups, meaning that more than one cluster could be randomised at each participating school. A computer-generated randomisation sequence was used to assign the clusters to the intervention and waitlist-control arms in a 1:1 ratio. Block randomisation of block sizes 4, 6, or 8 was generated in a computerised randomisation schedule. Randomisation took place after T1 data collection. The allocation sequence was concealed using an online central randomisation service set up and maintained by a professional third party (www.sealedenvelope.com), which concealed the sequence until group assignment. The randomisation process required a member of the research team (EI) to log into a password-protected website and enter the cluster ID code to receive the allocation. Randomisation outcomes were emailed to the research member conducting the randomisation (EI) and the trial manager (GW). Once allocated, neither project staff nor participants were blind to the assignment to groups.

### Intervention

The intervention arm was offered TRT [11], the group-based trauma support intervention detailed above and in the ASsIST trial protocol [25] directly after randomisation. In the main trial, the waitlist-control arm was offered TRT after the T3 data collection, approximately 20 weeks after randomisation.

### Data collection

Participant demographics, trauma history, and outcome data were collected using a secure online platform (Qualtrics). Trauma history was collected using the Refugee Trauma History Checklist [35]. Participants were also asked if anything unusually positive or unusually negative had happened during the study period. They were specifically asked if they had been informed of a family member or close person who had died or disappeared after they came to Sweden. In the mail trial, the primary outcome was child mental health, specifically symptoms of PTSD, depression, and anxiety. Secondary outcomes in the main trial included measures of emotional and behavioral difficulties, self-efficacy, and well-being, which relate to the TRT theory of change. Further details of the outcome measures are provided in the trial protocol paper [25].

### Assessment of pilot study outcomes

Participant demographics were derived from the questionnaire survey. The feasibility of randomisation was assessed using administrative records and demographic data from the questionnaire survey. The feasibility of screening, recruitment, attendance, and retention was assessed using administrative records. The suitability of the main trial outcome measures was derived from the questionnaire survey. The intention was to collect detailed attendance records and fidelity checklists; however, session-by-session attendance lists were not shared for any site, and the fidelity checklist was only provided for one site. Consequently, only basic attendance data (attendance at least one session) is reported, and intervention fidelity is not reported. We do know from correspondence with TRT group leaders that the caregiver sessions were omitted in one of the two delivering sites due to COVID-19 restrictions, one TRT group was postponed, and one TRT group was delivered at an accelerated pace to fit within the school term.

Intervention acceptability was assessed using qualitative interviews. Eleven semi-structured interviews were conducted by the first author (SLG) with accompanied children who had participated in the intervention but were not necessarily in the trial. They were recruited via TRT leaders. Apart from questions regarding perceptions, satisfaction, and experiences of participating in TRT, the interview guide also had specific questions regarding parental involvement, which were analysed and reported separately [36]. Follow-up questions were added during the interviews. An interpreter was present when needed. The interviews were considered to have provided rich enough data for our aim. Each participant was reimbursed with a gift card valued at approximately 200 SEK (≈ 15GBP).

### Analysis

Descriptive statistics for participant demographics, feasibility outcomes, and the main trial outcome measures were calculated using Microsoft Excel 2021, Version Office Professional Plus. Specifically, the mean and standard deviation of participant age were computed. The proportions of positive screenings, attendance, and retention were determined. Additionally, the mean scores and standard deviations for the main trial outcome measures at T1 and T2 were calculated.

**Fig. 2:**
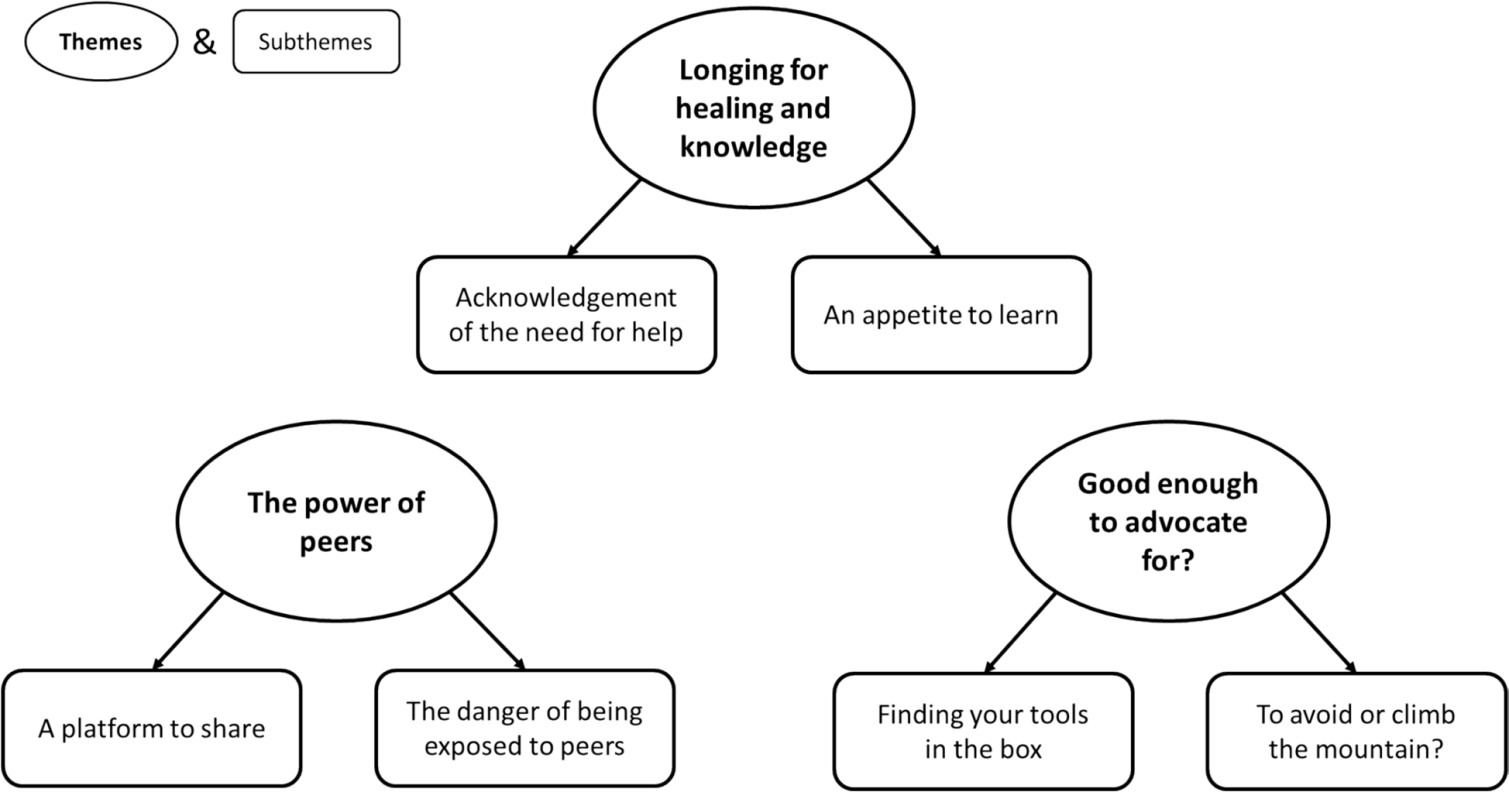
Overview of themes and subthemes

The interviews were transcribed and analysed by the second author (FA) using Thematic Analysis, which is a common method used in qualitative studies. As described by Braun and Clarke [37], Thematic Analysis requires researchers to perform a thorough analysis to describe and identify themes within the dataset. This process must follow specific and transparent steps to ensure trustworthiness and achieve rigorous results. The analysis process involved several steps: Familiarisation with the data, initiating preliminary codes by categorising text segments and refining them through multiple readings; identifying themes and sub-themes by analysing patterns in the coded data and removing overlapping or redundant codes, as well as refining themes; creating a thematic map (see Fig. 2), selecting quotes and assigning pseudonyms. Throughout the process, issues of trustworthiness were discussed between (FA), (SLG), and (GW).

The research team adopted the ADePT framework [34] when considering recommendations in light of the pilot trial. The ADePT framework provides a systematic way to generate and assess solutions to any issues that arise in pilot trials. The first step is to classify the issues into either: issues likely to be a problem only for the trial (Type A); issues likely to be a problem for both the trial and the real world (Type B); or issues likely to be a problem only for the real world (Type C). Second, solutions are generated by considering which aspects of the intervention, trial design, or context could be changed.

## Results

### Participant demographics

Twenty-two eligible children aged 10–18 years (M = 14.86, SD = 2.62) were randomised into five clusters (3 intervention, *n* = 11; 2 waitlist control, *n* = 11). Among the participants, seven were female and 15 were male. When asked for their ‘Nationality or homeland’, the participants reported: Afghanistan (*n* = 1), Congo (*n* = 2), Eritrea (*n* = 2), Ethiopia (*n* = 1), Iran (*n* = 1), Iraq (*n* = 1), Somalia (*n* = 5), Sweden (*n *= 1), Syria (*n* = 5), Thailand (*n* = 1), and Uganda (*n* = 2). Children chose to complete the self-report assessments in Arabic (*n* = 5), Dari (*n* = 2), English (*n* = 1), Tigrinya (*n* = 1), Swedish (*n* = 10), and Somali (*n* = 3). For those with available data (*n* = 13), around half (*n* = 8) had a residency permit, of which most (*n* = 5) were permanent and some (*n* = 3) were temporary. Demographics and descriptive statistics of participant trauma history are reported in Table 1.

**Table 1:** Demographics and potentially traumatic events for the intervention and control arms, presented as mean scores and standard deviations (SD), numbers and percentages (%)

Characteristic/event	Intervention *n* = 11	Waitlist (control) *n* = 11
**Age (M ± SD)**	12.73 ± 1.62	17.00 ± 1.34
**Sex (% M/F)**	55%/45%	82%/18%
**Nationality (*n*)**	Syria (4), Somalia (3), Eritrea (2), Uganda (2)	Congo (2), Iraq (1), Somali (1), Syria (1), Somalia (1), Iran (1), Thailand (1), Afghanistan (1), Ethiopia (1), Sweden (1)
**Potentially traumatic events (*n* (%))**		
*War at close quarters*	4 (67%)	7 (64%)
*Forced separation from family or close friends*	4 (67%)	7 (64%)
*Loss or disappearance of family member(s) or loved one(s)*	4 (67%)	7 (64%)
*Physical violence or assault*	4 (67%)	6 (55%)
*Witnessing physical violence or assault*	4 (67%)	7 (64%)
*Torture*	3 (50%)	7 (64%)
*Sexual violence*	1 (17%)	5 (45%)
*Other frightening situation where life felt in danger*	3 (50%)	7 (64%)
*Informed of death/disappearance after arrival in Sweden*	1 (17%)	2 (18%)

For the qualitative study assessing intervention acceptability, gender was balanced among the participating children; six identified as female and five as male. They were aged between 16 and 20 years (Mdn 18 years), and their country of origin varied: Syria (6); Palestine (2); Afghanistan (1); Kurdistan (1); and Ethiopia (1). In some cases, a period of up to two years had elapsed between participants’ engagement in the intervention and the subsequent interviews; hence, some youth had reached the age of 18 and above at the time of the interview.

### Feasibility of screening

In total, 171 refugee children were screened using the CRIES-8 [32]. Of these, 76 (44%) met the threshold for PTSD (i.e. scored 17 or above). Yet, 20 declined participation in the trial, and 34 did not meet the inclusion criterion of arriving in Sweden accompanied. As not all children were referred for participation, it is not possible to draw conclusions regarding the proportion who screened positive for PTSD against the original target. Of those referred *and* those screened via classroom, 44% screened positive for PTSD. See Fig. 1 for a flowchart of participants.

### Feasibility of recruitment

Screening commenced in October 2019. Two clusters, of three participants each, were randomised the following month. Two further clusters, of eight and four participants, were randomised in February and March 2020 respectively. There was a break in recruitment due to the COVID-19 pandemic. Then, a further cluster of four participants was randomised in November 2020. In total, 22 participants were randomised over 12 months, at which point the trial was stopped due to recruitment not being viable at the time. Therefore, the pace of recruitment was much slower than anticipated. The original target of recruiting 28 eligible children in the first 3 months was not met. The inclusion criterion specifying an age range of 8–17 years was not fully adhered to, as described under Trial Design.

### Feasibility of randomisation

The randomisation website functioned well. Four schools participated in the study, comprising a total of five distinct clusters formed based on established friendship networks within each school. The average cluster size (*n* = 4) was smaller than anticipated (*n* = 6). Three clusters were randomised to the intervention condition (cluster size 3, 4, and 4) and two clusters to the waitlist control (cluster size 3 and 8), which resulted in 11 participants in each trial arm (see Fig. 1 for the CONSORT participant flow diagram). The trial arms were imbalanced in terms of age, with the intervention arm (M = 12.73, SD = 1.618) younger than the waitlist-control arm (M = 17.00, SD = 1.342). The trial arms also appeared imbalanced in gender, with five females (45%) in the intervention arm and two in the waitlist-control arm (18%).

### Intervention attendance and trial retention

Research team administrative records indicated that the intervention attendance goal for at least 50% of those randomised to the intervention arm to attend at least one of the five core intervention sessions was achieved (*n* = 7/11) (64%). One intervention cluster (*n* = 4) did not receive the intervention as the intervention was postponed to the following school term. The trial retention goal for at least 50% retained at the T2 follow-ups was met (*n* = 20/22; 91%) and was balanced between the intervention (*n* = 10/11; 91%) and control (*n* = 10/11; 91%) arms.

### Main trail outcome measurement

The mean scores and standard deviations at T1 and T2 for the main trial outcome measures are reported in Table 2.

**Table 2:** Summary of outcome measures at T1 and T2 for the intervention and control arms, presented as mean scores and standard deviations (SD)

	**T1**		**T2**		
CRIES-13	*Intervention* *Control*	30.80 (9.48) 38.25 (16.02)	*Intervention* *Control*	18.80 (12.44) 26.44 (16.55)	
PHQ-9	*Intervention* *Control*	5.00 (4.64) 13.09 (9.32)	*Intervention* *Control*	6.29 (5.02) 9.33 (7.45)	
GAD-7	*Intervention* *Control*	5.83 (8.18) 8.91 (8.34)	*Intervention* *Control*	3.86 (3.98) 5.67 (4.27)	
SDQ	*Intervention* *Control*	9.17 (4.71) 14.18 (7.78)	*Intervention* *Control*	9.29 (7.50) 14.89 (8.70)	
Cantril Ladder	*Intervention* *Control*	7.82 (1.94) 6.09 (3.08)	*Intervention* *Control*	9.10 (1.85) 5.11 (3.22)	
GSE	*Intervention* *Control*	28.50 (7.61) 28.73 (7.61)	*Intervention* *Control*	31.57 (3.36) 25.67 (7.84)	

### Intervention acceptability

Three key themes emerged from the qualitative interviews: *Longing for Healing and Knowledge*, *Power of Peers*, and *Good Enough to Advocate For?* There were two sub-themes within each theme, as illustrated in Fig. 2.

#### Longing for knowledge and healing

This theme explores the factors that motivated participants to engage with TRT. The intervention was introduced by the TRT group leaders in a school setting, and its purpose resonated with children, who expressed a desire to acquire new tools to cope with mental health stress. Participants viewed the purpose of the intervention in different ways, some as a support resource for those impacted by war and adversity and some as a means to alleviate school-related stress. These motivations are further examined and categorised into the sub-themes: ‘Acknowledgment of the need for help’ and ‘An appetite to learn’.

##### Acknowledgement of the need for help

When introduced to the purpose of TRT, some children were already aware of the mental health issues they were facing. They described feelings of anxiety, a sense of hopelessness, stress, panic attacks, nightmares, and war-related flashbacks—issues they felt needed urgent support to help them settle in Sweden. They acknowledged the hardships of their journeys, sleep difficulties, and the impact this had on their well-being. Others expressed motivation to participate in the hope of reducing school-related stress.

*"You know we came from the war, so you feel you’d just like to talk about those things, which are hard to explain; it’s like flashbacks that come to your mind every day, every week, pictures that you would like to share with someone." (Amina, Palestine)*

##### An appetite to learn

Some children were motivated to attend TRT out of curiosity and a desire to learn new skills to manage life challenges. Although initially unfamiliar with the intervention’s structure and manual, the explanations provided by the TRT group leaders encouraged their involvement. They were eager to learn tools such as calming techniques for stress and creating an imaginary safe space for fear, which they found new and exciting. Some were also motivated by the group setting, which made the learning process enjoyable, as it felt collaborative and fun to explore these skills with friends.

*"Because I need this type of help, and I like to try all new things, everything that is new, I like to discover." (Ahmad, Syria)*

#### The power of peers

A sense of belonging was a repeated reason for the children to engage in TRT sessions. The children were cheerful about being in TRT and gathered with their friends, which motivated them to attend the sessions. They were excited about doing things together. At the same time, some children described feeling unsafe to share and distrust towards others. This theme consists of two sub-themes that explain the two sides of a coin: ‘*A platform to share’* and *‘The danger of being exposed to peers’.*

##### A platform to share

Some children highly appreciated the group setting and the TRT group leaders, who listened to their experiences in ways they had not found elsewhere, not even within their families. They described it as a safe space to express feelings and talk about their backgrounds without fear of judgment. For some, it was the first time they shared their experiences, and they found comfort in the support of their peers. One child recounted feeling speechless after hearing a friend’s difficult story but chose to offer support simply by listening. After attending TRT, participants found it easier to talk about their experiences. They were motivated by their peers’ stories, finding inspiration in how others handled challenges and recognizing their friends’ resilience. Hearing about others' hardships fostered a sense of connection, making them feel less isolated. The shared focus of the group helped create a sense of safety and belonging, which made it easier for them to express emotions. Some felt the TRT group leaders’ understanding and care enhanced this supportive environment.

*“The thing that you get to know is that you are not the only one who has problems in life; there are other people with problems, which makes you feel not alone.” (Abel, Ethiopia)*

##### The danger of being exposed to peers

On the other hand, some children felt unsafe being around new people, which caused discomfort and reluctance to share personal stories. They felt shame and feared that revealing private information could lead to betrayal or bullying if conflicts arose within or outside the group. For some, being surrounded by strangers heightened their sense of vulnerability, making them hesitant to express their emotions. Others disclosed they were unable to sustain friendships with those in the group after completing TRT; they were alone without any connections with the group or the leadership. Some group leaders reportedly addressed the relational issue by investing a lot of time in participants getting to know each other before starting to address their trauma symptoms, which was described as an essential aspect for one of the children.

*"I mean, I could maybe share with my boyfriend or close friend; the thing is, I feel alone and have a hard time trusting others." (Sozan, Kurdistan)*

#### Good enough to advocate for?

The descriptions concerning the children’s interaction and engagement with the techniques varied, with some participants finding the tools beneficial, while others described not experiencing the same value, which is explored in the first subtheme ‘Finding your tools in the box.’ The second subtheme ‘To climb or avoid the mountain’ explores the varied attitudes towards engaging in trauma-processing activities.

##### Finding your tools in the box

Some children found the techniques introduced in TRT beneficial and continued using them to manage stress and improve sleep after completing the intervention. They highlighted exercises such as listening to music, breathing techniques, and visualisation of a ‘safe inner place’ as valuable tools for managing anxiety, stress, and reminders of past traumatic events. Additionally, practices like positive thinking helped them combat feelings of hopelessness and depression. They also appreciated how TRT empowered them to communicate emotions to their families—an area they had previously struggled with.

*"Before I could not dare to express my feelings to anyone, now I can share them with my parents and siblings." (Abel, Ethiopia)*

However, some children were disappointed with some tools as they did not see the benefits of using them.

*“Some tools were useful, and some weren’t; if I would be honest, only one tool was useful for me, the stress exercise; the others were not useful.” (Ahmad, Syria)*

##### To climb or avoid the mountain?

This sub-theme refers to facing and addressing challenges, where children expressed different responses to confronting trauma. For some, the trauma processing journey felt like facing a daunting mountain, requiring a choice between avoiding or confronting the issue. Some children viewed participating in TRT as a way to confront and process past experiences to be able to focus on the future despite their suffering. They felt encouraged to share with friends what they had learned, emphasising that suffering would not last indefinitely. They conveyed that by processing their trauma, they could shift focus to future goals like adapting to life in Sweden, pursuing education, and aspiring to a career.

However, others found it more challenging to engage with their past and preferred to ‘move on’ by distancing themselves from painful memories, seeing starting over in Sweden as an opportunity to leave their past behind and avoid revisiting trauma. One child noted that her attendance at TRT sessions was primarily to support a friend rather than for her own needs, highlighting the diversity of motivations and coping strategies within the group.

*" I will explain that this was in our past. He needs to forget it and think of the new life in Sweden and that he is safe here."(Tarek, Syria)*

## Discussion

Overall, the internal pilot identified several feasibility issues, which warranted consideration in the context of pursuing the main trial (see Table 3).

**Table 3:** Feasibility issues and potential solutions identified using the ADePT framework

Problem type	Evidence	Potential solutions
**Type B** Lower than anticipated proportion of children screening positive for post-traumatic stress	Less than 50% screened above threshold on the CRIES-8	**Context** Enabling inclusive and sensitively conducted screening **Study design** Omit screening Set screening targets according to screening context
**Type B** Recruitment is inefficient	The recruitment goal of 28 eligible children was not reached	**Context** Convey intervention purpose in a culturally sensitive manner Be mindful of contributing to a deficit discourse and adopt strength-based approaches Be mindful of the refugee perspective when making trial decisions **Study design** Use cultural brokers Use strength-based recruitment materials for instance flyers for both site and individual recruitment Troubleshoot with refugee advisors Merge ASsIST and SUPpORT
**Type B** Defining and applying age eligibility criteria across trials presents methodological and ethical complexities	Age range violated; 18-year-olds included in pilot	**Context** Align trial age eligibility with the age range of the recruitment site (e.g. school enrollment ages) **Study design** Harmonise eligibility criteria across related trials Use automated eligibility checks and dual verification before randomisation Pre-specify how to handle participants who age out between screening and randomisation Provide more thorough protocol training and decision aids for staff Establish ethics review for borderline cases and document decisions transparently
**Type B** Few active sites	Low engagement of group leaders	**Context** Support TRT group leaders **Study design** Add supervision, experience meetings, peer-to-peer supervision
**Type A** Cluster randomisation caused demographic differences between intervention group and control group	Intervention and control groups were imbalanced in terms of gender and age	**Context** Pairing participants within clusters may be beneficial, as having a friend in the group can foster trust and a sense of safety **Study design** Consider including statistical correction for differences in baseline characteristics in the analysis plan

### Reflections on recruitment, screening, and reimbursement

In the current internal pilot study, the recruitment goal was not met. Refugee populations are often categorized as a ‘hard-to-reach population’ in research due to factors such as mobility, legal status, distrust of authorities, and linguistic or cultural barriers [38, 39]. However, this term risks oversimplifying the complexities of this group and may imply that the refugee population possesses intrinsic characteristics that may not only contribute to stigmatisation and othering but also remove the researcher’s responsibility in conducting culturally responsive recruitment [40]. In this study, the COVID-19 pandemic affected recruitment significantly; however, unlike many other countries that implemented strict lockdowns, Sweden opted to keep schools open for most children up to the age of 16 [41], and it is important to acknowledge that recruitment issues were present even before the pandemic. Addressing these challenges through the lens of cultural humility and “ethical reflexivity” (i.e. examining researchers’ values, assumptions, and actions to understand their ethical implications) renders some critical self-reflection. This discussion focuses on the ethical and practical dilemmas encountered during the trial, along with the valuable insights and lessons learned by the research team throughout the process.

Initially, recruitment was planned through multiple routes such as self-referral and referral via non-governmental organisations, as well as by schools, as described by Warner et al. [25]. However, as explained in more detail by Warner et al. [42], due to the rapid changes in Sweden’s political landscape, the adoption of restrictive immigration policies, and the dismantling of infrastructure previously established to meet refugees’ needs, the recruitment and the delivery of TRT were shifted solely to schools. Locating and engaging schools and TRT group leaders was difficult even prior to the COVID-19 pandemic, as some schools felt overburdened by their educational duty and did not hold the capacity to host the intervention or felt it was outside the scope of school provision. A deliberate change in the messaging used when contacting schools was adopted, as described in which, among other changes, the effect of mental health on learning was highlighted [43].

Similar to the SUPpORT feasibility trial [44], it was difficult to find active TRT locations despite having over 350 trained TRT facilitators in Sweden. The primary reason was a lack of capacity, stemming from insufficient funding for TRT delivery and/or an overwhelming workload from other responsibilities, which limited the time available to conduct TRT [44]. A qualitative study with group leaders revealed feelings of inadequacy and a lack of support and supervision, especially when faced with children expressing suicidal thoughts [45]. This could be yet another reason for group leaders not engaging in TRT despite having been trained. A review of the implementation of mental health interventions in schools highlights the importance of ongoing supervision and support from leadership within schools [46]. In an RCT conducted in schools in Gaza, the TRT intervention was supported by the Ministry of Education, and group leaders received weekly supervision [16]. The importance of supervision for TRT group leaders is stressed by Heltne et al. [47] with an additional purpose to reduce the risk of secondary traumatisation. Furthermore, one may speculate if Sweden’s historical shift in immigration policies and the growing anti-immigrant discourse [48] may have affected TRT leaders’ support, possibilities, and motivation to engage in various ways.

It was not possible to assess the initial target of achieving a 50% positive screening rate. Naturally, this percentage depends on the setting where screening occurs; refugee children preselected by school counsellors or those who self-referred may screen positive to a higher degree compared to those universally screened in a classroom setting. In the current study, the intention was for children to be referred. However, the majority were screened in a classroom setting.

While it has been suggested that schools’ informal environments foster trustful relationships and provide an ideal context for addressing the mental health needs of refugee children [9], screening for trauma symptoms in a classroom setting warrants careful consideration. Such practices may risk labelling students, particularly when individuals scoring above a certain threshold are singled out for participation in interventions, potentially affecting their relationships with peers and teachers. Furthermore, it is important to recognise that schools may not represent safe environments for all refugee children, as some may encounter rigid rules, suspicion, hostility, and discrimination from both teachers and peers [49], which could potentially deter them from disclosing symptoms when screened in a classroom setting.

Regardless of the context, using the screening of post-traumatic stress symptoms as an inclusion criterion for TRT renders reflection in itself. RCTs conducted in the Global South have adopted varying screening procedures [14, 15] and sometimes have chosen not to screen [16]. In retrospect, a similar approach might have been optimal for the RCT in Sweden. Omitting the screening requirement would have significantly eased recruitment efforts, enabling greater numbers of children to access the intervention in a trial setup. However, it is important to consider potential adverse effects for children without PTSD symptoms attending TRT groups, for instance, the risk of secondary traumatisation.

Moreover, in retrospect, dividing the accompanied children and unaccompanied children into separate trials may not have been the optimal choice, not only from a recruitment perspective but also from an ethical perspective. In literature and public discourse, unaccompanied refugees are repeatedly described as the most vulnerable, failing to acknowledge the resilience, agency, and diversity within this group [50]. Some children may also transition between being unaccompanied and accompanied due to family unification and separation during the process of migration. The separation of accompanied and unaccompanied children may have inadvertently reinforced a discourse that divides these groups, potentially widening the gap between them by overlooking that these groups have more similarities than differences, considering the refugee experience and the post-migratory conditions [51]. It is also important to consider the social aspects of separating these children, for instance, when accompanied and unaccompanied refugee children, who may be in the same class or have formed friendships, wish to attend TRT together on the same premises but are hindered from doing so. In the parallel RCT (SUPpORT) [24] involving unaccompanied youth, the eligibility criteria included individuals aged 14 to 20 years, whereas the main trial for this pilot was designed for participants aged 8 to 17 years. Interestingly, this age boundary was not strictly followed in the pilot, as some 18-year-olds were randomised into the study. This deviation invites reflection on several possible contributing factors. It is conceivable that some participants reached the age of 18 years between the initial screening and the point of randomisation. Alternatively, the research team member conducting the randomisation may have inadvertently applied the criteria from the parallel trial, or perhaps made a deliberate ethical judgment, feeling it was unjust to exclude students who had just surpassed the age threshold.

Another point to consider when recruiting is the potential risk of coercion as refugees are more likely to experience structural coercion [52]. To minimise the risk in this study, we consistently emphasised the voluntary nature of participation and assured participants that their choice to decline or withdraw from the intervention would not result in any negative consequences. We also communicated that joining the intervention wouldn’t lead to any advantages in the asylum-seeking process. However, considering the financial constraints faced by refugee children in Sweden (daily allowance may be as low as 12 SEK (≈1 GBP) for some children) [53], the reimbursement of 200 SEK (≈ 15 GBP) for completing screening questionnaires or interviews may, in retrospect, have been excessive as there is a risk the amount would pressure some refugee children to participate to meet their immediate needs and not due to a genuine will to participate. This concern was deliberated within the research team but was not extended to discussions with public advisors. We ultimately determined that offering varying compensation to different participant groups would be unethical. Consequently, participant groups within our broader research project, i.e. TRT leaders, refugee parents, and children, were all offered the same level of reimbursement.

Future research would benefit from a reflective approach on these matters early on in the research process and from incorporating insights from children with experience of forced migration in this reflection.

### Outcome measures

The baseline levels of post-traumatic stress symptoms, as indicated by the CRIES-13, aligned with expectations based on previous TRT studies in Sweden [13, 44], albeit the former studies were conducted with unaccompanied youth rather than accompanied children. The fact that the CRIES-8 was utilised to screen children for participation in the trial also leads to the assumption that baseline CRIES-13 scores would be high. It appears that the CRIES-13 is sensitive to change, indicating its suitability as an outcome measure. The Cantril Ladder also appears to be sensitive to change and has the advantage of being an easy-to-complete single-item measure, which has been shown to satisfactorily indicate life satisfaction among refugee children and youth [54]. The GSE [55] seemed somewhat sensitive to change, yet it is interesting to note the high levels of self-efficacy at baseline. The suitability of the PHQ9 [56], GAD-7 [57], and SDQ [58], on the other hand, warrants further consideration as there was no indication of sensitivity to change. However, it is not possible to draw conclusions regarding the suitability of the measures from the current study, given both the small sample size and that fidelity and detailed attendance data were lacking. According to administrative records, 64% of those randomised to intervention attended at least one session. While this met the operational feasibility criterion, defined as at least half of the randomised children attending one session, there is currently no available evidence on dose–effect responses for TRT. Therefore, it remains uncertain what minimum number of sessions is required to achieve therapeutic effectiveness. On reflection, it is unlikely that attending only one session would be sufficient to produce meaningful clinical outcomes.

### Engagement and intervention delivery

The term ‘adherence’ is frequently used to describe participants’ level of engagement in an intervention. However, this terminology may be problematic, as it may carry a judgemental undertone [59], and in this setting, using ‘adherence’ risks casting refugee children in a passive role, overlooking the complexities of their individual experiences, motivations, and legitimate reasons for choosing not to engage in the intervention. Although the intention was to collect detailed attendance records, these were not returned by TRT leaders; hence, it is not possible to provide information on whether the children attended every session.

Due to one site postponing the intervention delivery to the following semester, only two of the three sites randomised to the intervention actually delivered the intervention. At one intervention site, sessions were delivered at a faster pace than originally planned to align with the school’s scheduled holidays. Moreover, in one of these sites, T2 data collection did not take place because the children were not attending school. Using schools as an intervention setting can pose logistical challenges. A review on implementing mental health intervention at schools identified several such barriers, suggesting that practitioners delivering interventions in school settings must recognise and be prepared to navigate the limitations imposed by school schedules and timetables [46].

School engagement of refugee parents should also be considered. A qualitative study conducted in Sweden revealed that TRT group leaders were not fully equipped to manage practical challenges in reaching refugee parents and harboured concerns about refugee parents’ readiness to engage in the intervention, leading some to not invite refugee parents at all [36]. In the current study, the caregiver sessions were omitted in one of the sites; however, this was reportedly due to COVID-19 restrictions prohibiting parents from gathering.

### Reflections on the acceptability of intervention

The children expressed motivation for the intervention, valuing tools aimed at managing mental health and life skills. Their interpretations of the intervention purpose varied, with some focusing on stress reduction and others on broader mental health and trauma support. Some appreciated the group format, especially when trusting relationships were present, as it fostered mutual support and shared experiences. However, the complex dynamics with peers in the TRT group were also highlighted. While peers could provide significant support, some children expressed apprehension about sharing personal stories, fearing that sensitive information might later be misused, particularly in the event of a fallout. A recent systematic review of qualitative findings on stigma in school-based mental health interventions (in a non-refugee specific population) highlights challenges within the school environment, such as the proximity to peers, pre-existing social hierarchies, the experience of ‘negative labelling’ by peers, discriminatory reactions such as bullying by peers and concerns about ‘compromised confidentiality’. The study also described ‘restricted disclosure’ due to mistrust and distancing from support was acknowledged [60]. This demonstrates that also within non-refugee populations, the stigma surrounding mental health remains prevalent. Peers may play a supportive role, but can also present a barrier to full participation in mental health interventions placed in schools. Hence, this experience is universal and should not be framed as unique to refugee children.

Participants showed particular enthusiasm for techniques such as stress management and creating a ‘safe inner place’, which they found novel and helpful. The collaborative group setting further enriched their engagement and enjoyment of the learning process. Despite the added ‘getting to know each other’ session in the Swedish version of TRT, they emphasised the importance of facilitators building rapport and understanding individual interests before addressing specific issues. Since trauma exposure, PTSD, and the refugee context may lead to feelings of distrust [61], it is crucial that facilitators actively mitigate this, build a safe, confidential environment, and consider strategies that allow children to feel more secure in sharing. Further, the children shared that although TRT sessions were considered helpful, not dealing with traumatic memories and ‘just moving forward’ was also perceived as a healing option. This is in line with previous research on avoidance coping and trauma [62] as well as findings from TRT in the Global South where issues of creating safe environments, non-disclosure, and avoidance of trauma memories were raised by the TRT group leaders in supervision [47].

### How TRT is presented to children

The qualitative findings indicated varied motivations among children for joining the TRT group—some emphasised the need for tools to address specific mental health problems and trauma symptoms, while others sought broader strategies to manage stress. This variation may reflect the evolving presentation of TRT over time.

Initially, TRT was introduced to children as an intervention to potentially reduce trauma symptoms and post-traumatic stress; however, after feedback from one of the sites with successful recruitment using cultural brokers, TRT was presented as a ‘course’ that might help with stress, concentration difficulties, and sleep and, although trauma was mentioned, it was not in terms of PTSD. This is in line with Miller et al. [63] who recommend a strengths-based approach when communicating with refugee children and suggest framing mental health problems in terms of stress. Similarly, Gronholm et al. [57] suggest that personal agency is particularly important during adolescence and is a key consideration when designing mental health interventions and recommend framing mental health interventions in terms of stress reduction or life skills. A recent qualitative paper summarising twenty years of experiences of training TRT leaders in the Global South suggests similar recommendations concluding that terms such as ‘trauma reactions’ and ‘post-traumatic stress’ require modification and adaptation, not only due to local terminology for trauma and traumatisation being associated with psychiatric illness and carrying a stigma but that in some places these terms might be unfamiliar and lack any meaningful resonance. At the same time, they stressed the importance of not downplaying the more challenging trauma-focused part of TRT, as it appears that some TRT leaders presented TRT as a group to ‘have fun’ in order to motivate participation [47].

The initial presentation of TRT in Sweden, including the promotional posters and oral descriptions of the intervention in schools, may have unintentionally contributed to a deficit-oriented reductionistic perspective on refugee children. By heavily emphasising symptoms of PTSD and trauma related to migration, this approach risked framing participants solely through the lens of trauma. It may also have limited relatability, as it overlooked the broader context of the children’s daily challenges, such as navigating cultural gaps within their families, dealing with discrimination, and facing the complex stressors associated with resettlement [51].

In the present study, the Refugee Trauma History Checklist [35] identified a high prevalence of trauma exposure among participants, including experiences such as being in close proximity to war, witnessing or experiencing physical violence or assault, and enduring the loss of loved ones. However, when asked about recent negative life events, the children predominantly mentioned typical children’s concerns such as breaking up with a boyfriend or conflicts with their parents. Furthermore, despite adversities, the baseline scores on the GSE [55] were surprisingly high, which may be a sign of resilience or post-traumatic growth. Chase et al. [64] explore the ethical and methodological dilemmas researchers face when studying the experiences of refugee children and caution against an over-emphasis on migration trauma, which risks reducing children’s identities to their refugee status and obscuring other essential aspects of their lives. Instead, they argue that refugee children should be seen holistically—as young people with their own aspirations, dreams, and complexities beyond their migration experiences. Chase and colleagues also examine the delicate balance between recognising the agency and the vulnerability of refugee children. They highlight the complexity of assigning vulnerability and victimhood solely to make refugees appear ‘deserving’ of protection, potentially reinforcing disempowering narratives and overshadowing resilience [64]. In-depth interviews with refugee children in Sweden confirm that, despite challenging experiences, the main narrative was *not* identifying as a victim and having high aspirations in life [65]. These aspects of vulnerability, agency, and recognition of normal developmental struggles need to be navigated when introducing a mental health intervention to these children. Not only should trauma interventions be presented in a strength-based manner, without diminishing mental health struggles and framing them in a systemic perspective, but they should also be culturally responsive, acknowledging that refugees are a heterogeneous group with many cultural backgrounds [66].

### Patient and public involvement

As described in the ASsIST trial protocol [25], the trial design was informed by refugee advisors. Patient and public involvement (PPI) such as this is increasingly recognised in research; however, conducting PPI in a respectful and culturally sensitive manner, while addressing relational power imbalances, can be challenging and requires significant time and careful deliberation [67]. The implementation of PPI within TRT in Sweden, described by Lampa et al. [68], provides valuable learning experiences. When TRT was introduced in Sweden, the research team, who had limited experience of PPI, involved refugee parents as advisors in the steering committee for the RCT. The advisors offered valuable insights into the circumstances for refugees in Sweden as well as useful contributions to the planning of the RCT. However, there were issues in the involvement process; the advisors were not given sufficient time or preparations for discussions, nor were they provided with the context needed to fully understand the scope of the work. At the time, the team lacked the knowledge to establish mutually trusting relationships with the advisors. In the later stages of the RCT, the advisors revealed that they had not trusted the research team in the earlier stages. Despite good intentions, the collaboration with refugee advisors, in retrospect, remained at a superficial level. Hence, there were missed opportunities to gain valuable insights into culturally sensitive recruitment and intervention delivery issues. Future studies can benefit from working together with refugee advisors early on in the research process, supported by the emerging evidence and guidance on ethical and meaningful PPI with refugees [67, 69].

### Limitations

Beyond the issues already discussed, a remaining uncertainty about feasibility is the lack of returned fidelity checklists and attendance registers. Hence, we cannot report on intervention delivery or participant engagement at a session-by-session level, which is a major limitation. Moreover, it is important to reiterate the context in which this pilot study was conducted, with policy changes and the COVID-19 pandemic taking place during the study period. Significant societal shifts such as these complicate the generalisability of the pilot study findings to a larger trial; however, restrictive immigration policies persist, and the multitude of ethical and practical dilemmas experienced during the pilot study offer valuable insights into the challenges and considerations involved in conducting research with refugee children.

## Conclusions

Although the national RCT was not deemed feasible, there are many lessons to be learned for future mental health interventions addressing the needs of refugee children. Adopting a strength-based approach when introducing a mental health intervention to refugee children may not only reduce stigma and increase relatability, but it may actively shift from the deficit-focused discourse often applied to them. Although schools may be hesitant to host mental health interventions, framing these interventions as tools that could potentially support academic achievement may increase their willingness to participate. Peer support from those with similar experiences can promote normalisation, validation, and a sense of belonging, which are particularly beneficial for trauma-affected children. However, it is crucial to be mindful of existing social dynamics and hierarchies within school settings, as these may hinder full participation in group-based interventions. Screening for trauma symptoms requires careful consideration, both regarding the appropriateness of the methods used, ensuring the process is safe, respectful, and non-stigmatising, and whether such screening is necessary depending on the goal of the intervention. Separating accompanied and unaccompanied children in research trials may be problematic. Furthermore, defining and applying age eligibility criteria across trials presents practical and ethical complexities. Recent progress in working with PPI in research is promising, but building trust and involving refugees early in the research process is essential for their meaningful involvement. Cultural humility and sensitivity are fundamental in this process, as they could foster trust and ensure that interventions are respectful, relevant, and responsive to the needs and perspectives of refugee children.

## Supplementary Information

Supplementary Material 1.

## Data Availability

The data are not publicly available due to ethical restrictions. The small data set contains information that could compromise the privacy of research participants. Requests to access the data should be directed to the corresponding author.

## Abbreviations

ADePT: A process for Decision-making after Pilot and feasibility Trials
ASsIST: Accompanied refugeeS In Sweden Trial
CRIES: Children’s Revised Impact of Event Scale
GAD-7: Generalized Anxiety Disorder-7
GSE: General Self Efficacy Scale
PHQ-9: Patient Health Questionnaire-9
PTSD: Post-traumatic stress disorder
RCT: Randomised controlled trial
RTHC: Refugee Trauma History Checklist
SDQ: Strengths and Difficulties Questionnaire
TF-CBT: Trauma-Focused Cognitive Behavioural Therapy
TRT: Teaching Recovery Techniques
